# Clinical Analysis of Repeat Penetrating Keratoplasty in Children

**DOI:** 10.3390/jcm12093346

**Published:** 2023-05-08

**Authors:** Yajie Sun, Qi Lin, Peng Song, Xu Li, Zhiqiang Pan

**Affiliations:** 1 Department of Ophthalmology, Henan Provincial People’s Hospital, Henan Eye Institute, Zhengzhou 450003, China; 2 Department of Ophthalmology, Beijing Children’s Hospital, Capital Medical University, National Center for Children’s Health, Beijing 100045, China; lcat2002@163.com; 3 Beijing Ophthalmology & Visual Science Key Laboratory, Beijing Tongren Eye Center, Beijing Tongren Hospital, Capital Medical University, Beijing 100005, China; alicina7777@163.com

**Keywords:** repeat penetrating keratoplasty, children, graft survival

## Abstract

(1) Background: To analyze the indications, graft survival, and graft failure-related risk factors of repeat penetrating keratoplasty (RPK) in children. (2) Methods: In this case series, children younger than 12 years who received RPK at Beijing Tongren Hospital were reviewed. The indications for RPK, postoperative complications, and graft survival were analyzed. The analysis of the potential variables associated with graft survival was performed using Cox proportional hazards regression. (3) Results: A total of 30 RPK eyes of 29 children were included in this study. The mean follow-up time was 26.98 ± 18.75 months. The most common indication for RPK was a vascularized corneal scar (86.67%). Postoperative complications occurred in 27 eyes (90%), including immune rejection (46.67%), epithelial defects (36.67%), and glaucoma (26.67%). About 60% of the regrafts remained clear one year after RPK, while the overall graft survival rate was 30% at the last visit. The most common cause of regraft failure was irreversible immune rejection (8/21). The significant risks of graft failure included an age of less than 60 months at surgery (*p* = 0.009), corneal vascularization (*p* = 0.018), and a postoperative epithelial defect (*p* = 0.037). (4) Conclusions: A vascularized corneal scar is the most common indication of RPK in children. Immune rejection is the most prevalent complication, and irreversible immune rejection always causes regraft failure.

## 1. Introduction

Pediatric keratoplasty was performed infrequently before the mid-1970s [1]. With the improvement of surgical techniques and postoperative management, pediatric keratoplasty is becoming popular, and the age at surgery is becoming younger. However, pediatric keratoplasty is still recognized as a high-risk keratoplasty due to difficulties in preoperative and postoperative evaluation, low scleral rigidity, high vitreous pressure, and severe postoperative inflammation [2,3]. The high rejection rate and irreversibility of penetrating keratoplasty (PKP) in children may lead to low graft survival, so repeat penetrating keratoplasty (RPK) is quite often required [4,5,6]. In addition, the restoration of the normal visual pathway is important for visual development and facial development in children, even when regraft survival is dismal [6].

Previous studies indicated that the graft survival rate of RPK was lower than that of primary penetrating keratoplasty (PPK) in children. Xie et al., reported that the graft transparency rate after PPK in children was 74.3% but decreased to 38.1% after RPK [7]. Yang et al., also revealed that the graft survival rate after RPK (6%) was significantly lower than that after PPK (36%) in eyes with Peters anomaly [8]. However, few studies have specifically investigated RPK in children. We could not obtain enough detailed information about indications, postoperative complications, and graft failure-related risk factors after RPK in children. Numerous clinical studies on RPK in adults have provided a wealth of valuable information to help clinicians predict the postoperative effect, manage periprocedural complications, and improve graft survival [9,10,11]. Our previous study reported that the graft failure of RPK was higher (52.0%) than that of PPK (28.1%) in infant and child patients with corneal opacity [12]. In the current study, we focused on RPK in children and analyzed the indications, graft survival, associated prognostic factors, postoperative complications, and management after RPK in children in order to help clinicians and parents select an appropriate strategy and achieve better results.

## 2. Materials and Methods

### 2.1. Patients

This case series study was approved by the Medical Ethics Committee of Beijing Tongren Hospital, which is affiliated with Capital Medical University, and it adhered to the tenets of the Declaration of Helsinki. The medical records of children under 12 years of age who had obtained RPK at Beijing Tongren Hospital between December 2013 and June 2019 were reviewed. The included patients were followed up for at least 12 months unless regraft failure occurred within 12 months.

The following items were recorded: the patients’ demographics (age, sex, and laterality at surgery); systemic abnormalities (developmental delay, congenital heart disease, and cheilopalatognathus); the indications for PPK and RPK; the interval time between PPK and RPK; the intraocular pressure, lens status, and corneal vascularization of the recipients before RPK; the associated ocular surgeries performed concurrently or subsequently to RPK; and the graft diameter of the donor, follow-up duration, graft survival time after RPK, postoperative complications, and graft clarity status after RPK at the last visit.

Graft failure is defined as the irreversible loss of clarity. The graft survival time is defined as the time between the date of surgery and the date of corneal clouding onset or the date of the last visit [13].

### 2.2. Surgical Procedures, Postoperative Care, and Complication Management

Routine PK techniques and postoperative medication regimens were performed, as described in a previous study [14]. All the RPK surgeries in this study were performed by Zhiqiang Pan. The postoperative medication included glucocorticoids (prednisolone acetate ophthalmic suspension was used every 4 h, tobramycin and dexamethasone eye ointment were used every night for the first few weeks, and then fluorometholone 0.1% eye drops were used until at least 24 months after surgery), topical calcineurin inhibitors (tacrolimus 0.1% eye drops were used until at least 24 months after surgery), and anti-infection eye drops (until all the sutures were removed). In the cases with complications, different management methods, such as medicine or surgery, were performed according to the type and severity.

### 2.3. Statistical Analysis

Statistical analyses were carried out using IBM/SPSS software version 21 (IBM/SPSS Inc., Chicago, IL, USA). Descriptive statistics were reported. The survival of the regrafts was analyzed using the Kaplan–Meier survival method. Analysis of the potential variables associated with graft survival was performed using Cox proportional hazards regression. *p* < 0.05 was considered statistically significant.

## 3. Results

### 3.1. Patient Cohort

A total of 30 RPK eyes of 29 patients were included in the current study, and all of them were initial RPK. One patient received bilateral RPK, and the other 28 patients received unilateral RPK. The average age of the patients at the time of RPK was 59.18 ± 31.16 months. Surgery was performed in 19 eyes at the age of 60 months or less and in 11 eyes at the age of more than 60 months. Female patients accounted for 33.33%. The average time interval between PPK and RPK was 29.08 ± 23.44 months, and the average follow-up time after RPK was 26.98 ± 18.75 months.

### 3.2. Indications and Surgical Procedures

**Figure 1:**
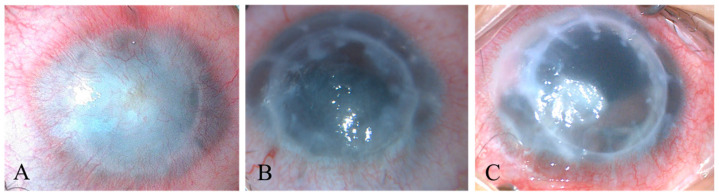
The Anterior Segment Photograph of Children with Different Indications of RPK. (**A**): Vascularized corneal scar; (**B**): Bullous keratopathy; (**C**): Corneal ulcer.

The indications for primary and repeat penetrating keratoplasty are summarized in Table 1. The anterior segment photographs with different indications for RPK are shown in Figure 1. The most common indication for PPK in children was congenital corneal opacity (CCO) (86.67%). Among them, Peters anomaly was the most common (53.33%), followed by unclassified CCO (16.67%), a scleral cornea (13.33%), bullous keratopathy (6.67%), congenital aniridia (3.33%), a corneal ulcer (3.33%), and post-traumatic leukoma (3.33%). The most common indication for RPK was a vascularized corneal scar (50%), which was secondary to graft rejection, suture loosening, a corneal ulcer, and anterior synechia of the iris. The other two indications for RPK were a corneal ulcer (26.67%) and bullous keratopathy (23.33%).

Furthermore, nine eyes (30%) underwent concurrent procedures at the time of RPK, including extracapsular cataract extraction (four eyes), cyclocryotherapy (two eyes), iridocyclectomy/pupilloplasty (two eyes), and anterior vitrectomy (one eye). Subsequent intraocular surgeries were performed in seven eyes (26.67%), including extracapsular cataract extraction and/or intraocular lens insertion and/or anterior vitrectomy (four eyes), anterior chamber irrigation (one eye), anterior vitrectomy and retro-corneal membrane resection (one eye), and cyclocryotherapy (one eye).

### 3.3. Postoperative Complications and Management

Postoperative complications occurred in 27 eyes (90%) after RPK (Table 2). A total of 13 eyes had a single complication, and 14 eyes had two or more complications. In addition, six eyes had clear grafts after timely and proper management, in which five eyes had single complications and one eye had compound complications. Furthermore, 14 eyes experienced immune rejection, including 4 eyes with simple rejection and 10 eyes complicated with other complications. Epithelial defects were found in 11 eyes; one eye with a simple epithelial defect and one eye complicated with a cataract were treated using a bandaged contact lens, and the two eyes had clear grafts. The other nine eyes progressed to a persistent epithelial defect or corneal erosion, even perforation, and the epithelium healed after symptomatic treatment, but the grafts failed and became cloudy. Glaucoma occurred in eight eyes; one eye with single glaucoma had a clear graft, and the other seven eyes complicated with other complications had failed grafts. A cataract occurred in seven eyes (three eyes with a simple cataract and four eyes complicated with other complications). Two eyes with a simple cataract underwent extracapsular cataract extraction surgery, and one of them had a clear graft. Two eyes that were complicated with other complications underwent surgery and had failed grafts. In addition, graft failure occurred in one eye of the other three eyes without cataract surgery. One eye had a corneal ulcer and failed. A retro-corneal membrane appeared in two eyes. One eye underwent an anterior vitrectomy and a retro-corneal membrane resection and failed due to rejection; the other one without surgery failed due to glaucoma and rejection. Hyphema occurred in two eyes, and the grafts failed.

### 3.4. Graft Survival, Causes, and Related Risk Factors of Graft Failure

**Figure 2:**
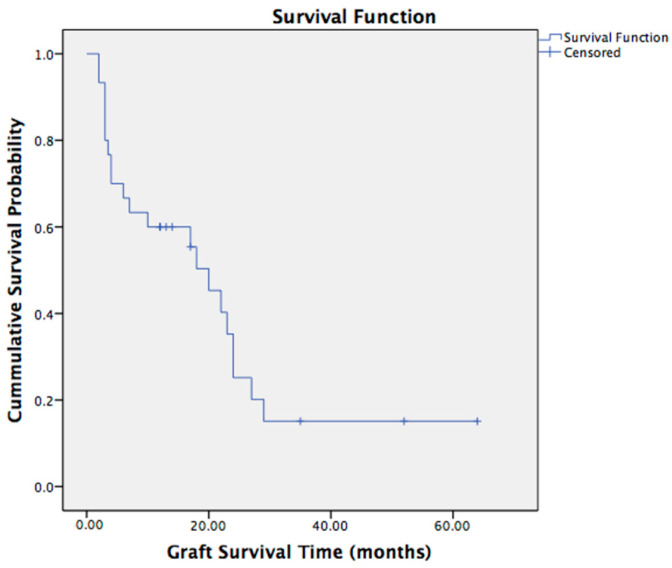
Kaplan–Meier Analysis of Graft Survival of RPK in Children.

At the most recent follow-up, nine (30%) regrafts were clear. The Kaplan–Meier graft survival curve is shown in Figure 2. The Kaplan–Meier graph demonstrated two big leaps of survival. One was within the first year, strictly speaking in the first 6 months (the probability of the regraft remaining clear was 66.7% at 6 months and 55.4–60% at 1 year), and the other one was within the second year (the probability of the regraft remaining clear was 25.2% at 2 years). The predicted mean survival time was 21.55 ± 4.09 months. The median survival time was 20.00 ± 3.315 months.

**Figure 3:**
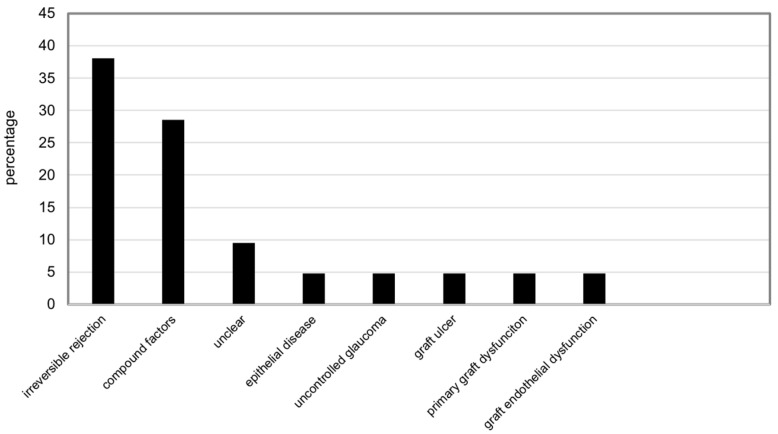
Regraft Failure Causes Distribution.

Of the 21 failed regrafts, irreversible immune rejection was the most common cause (eight eyes), followed by compound factors (six eyes), unclear factors (two eyes), epithelial disease (one eye), uncontrolled glaucoma (one eye), a graft ulcer (one eye), primary graft dysfunction (one eye), and graft endothelial dysfunction (one eye) (Figure 3). The details of the six regraft failure cases caused by compound factors included the following: three cases were caused by immune rejection and uncontrolled glaucoma, two cases were caused by epithelial disease and uncontrolled glaucoma, and one case was caused by immune rejection and epithelial disease.

The Cox regression analysis of the potential risk factors affecting the graft survival showed that the significant risks of graft failure included an age of less than 60 months at surgery (HR: 5.210, 95%CI: 1.505–18.036, *p* = 0.009), corneal vascularization (HR: 3.793, 95%CI: 1.257–11.444, *p* = 0.018), and a postoperative epithelial defect (HR: 3.527, 95%CI: 1.078–11.543, *p* = 0.037) (Table 3).

## 4. Discussion

RPK is quite often required as there is a high probability of initial graft failure in children. Previous studies have analyzed the indications, graft survival, complications, and prognostic factors affecting graft failure of RPK in adults [9,11,15], but few studies focused on children. Here, we comprehensively analyzed the indications, graft survival, postoperative complications, and related risk factors of RPK failure in children. The results showed that a vascularized corneal scar was the most common indication of RPK. Immune rejection was the most common postoperative complication and the most common cause of regraft failure. The overall graft survival rate was 30%, and the significant risk factors affecting graft survival were age at surgery, corneal vascularization, and a postoperative epithelial defect.

For the children who underwent RPK in this study, the most common indication for PPK was CCO. On the one hand, CCO is the most common indication for PPK in children in developed countries, even in China [7,12,16,17]. On the other hand, the graft failure rate is higher in children with CCO than in those with acquired corneal opacity, leading to a higher proportion of regrafts [12,18].

A previous study, which included 279 repeat keratoplasty of 219 adults from 1991 to 2017 in New Zealand, showed that the most common indication for repeat keratoplasty was endothelial decompensation (37.6%) [9]. In another study, which included 149 regrafts of 105 eyes in Turkey, the most common primary indication for RPK also was bullous keratopathy (31.4%) [10]. However, in this study, a vascularized corneal scar was the principal indication (50%) for RPK in children. The difference in the indications for RPK between children and adults may be explained by several aspects. First, CCO, such as Peters anomaly and scleral cornea, as the main indication for PPK in children are always accompanied by corneal vascularization [19]. In the current study, 36.67% (11/30) of the children who underwent RPK had neovascularization before PPK. Second, the corneal wounds of children healed much faster, and if the sutures were not removed in a timely manner, neovascularization episodes were incited [20]. Finally, graft rejection as the main cause of graft failure in children can induce corneal neovascularization or a vascularized corneal scar [21]. In this study, neovascularization after PPK was secondary to graft rejection, suture loosening, corneal ulcers, and anterior synechia of the iris.

Previous studies have reported that the graft survival rate of RPK in children varies from 6% to 67% [1,8,22]. In this study, about 60% of regrafts remained clear 1 year after RPK, while the overall graft survival rate was 30% at the last visit. Compared with our previous studies, the graft survival rate of RPK in children was significantly lower than that of PPK in children (71.85%) [16]. Theoretically, the lack of anterior chamber-related immune deviation after RPK would lead to high immune activity and a high risk of allograft rejection [23].

Corneal vessels can destroy the local immune privilege, increase the possibility of rejection, and then cause graft failure [12,24]. In addition, corneal vascularization may increase the risk of postoperative epithelial defects and graft opacity due to limbal stem cell deficiency [25]. Our results showed that the survival rate of regrafts with corneal vascularization was lower than those without vascularization. Although there are various methods to treat corneal vascularization, such as laser/phototherapy, topical anti-inflammation agents, subconjunctival injection of anti-vascular endothelial growth factor (VEGF) agents, and limbal stem cell transplantation, the vascularization often relapses, and the safety of these treatments in children is uncertain [23,26]. More studies on anti-corneal vascularization in children are needed to improve the graft survival of RPK.

A previous study reported that infants (<5 years) exhibited poorer graft survival than children aged older than 5 years [27], which is in line with our results in this study. Meanwhile, our previous study (including only patients under 5 years old) revealed that children older than 24 months who underwent PPK had poorer outcomes than those younger than 24 months [28]. Therefore, we considered that the optimal age ranges for penetrating keratoplasty in children may be younger than 24 months or older than 5 years. However, to verify this hypothesis, more detailed and powerful evidence is needed.

A postoperative epithelial defect is another risk factor for regraft failure in RPK children (*p* = 0.004), which is consistent with that in RPK adults [11]. As the main indication of RPK, vascularized corneal scars are often accompanied by abnormal limbal stem cells, which increase the risk of postoperative epithelial defects. If an epithelial defect is not repaired in a timely manner, more severe complications, such as graft opacity, infection, and corneal perforation, will occur. The bandaged contact lens (BCL) has been confirmed to be effective in the treatment of a persistent epithelial defect (PED) by preventing blinking-associated mechanical stress, especially in patients with ocular surface disease [29,30]. In this study, we used the BCL to treat epithelial defects and obtained good effectiveness. The BCL was used immediately after epithelial defects were found in two eyes, and the grafts remained clear at the last visit. We suggest that BCL be used as soon as possible once a corneal epithelial defect occurs.

## 5. Conclusions

In general, we presented some data on the indications, graft survival, complications, and risk factors of RPK in children. The most common indication for RPK was a vascularized corneal scar. These patients are prone to postoperative complications due to the abnormality of limbal stem cells and the local immune status. In addition, due to the poor cooperation of children, it is difficult to find, identify, and handle postoperative complications, which leads to a low graft survival rate. Immune rejection was the most common postoperative complication and the most common cause of regraft failure. The risk of graft failure was higher in children with an age of less than 60 months at surgery, preoperative corneal neovascularization, and postoperative epithelial defects. Pediatric keratoplasty is relatively rare due to the low incidence of corneal disease in children. Additionally, the high costs of keratoplasty and the lack of donors result in a smaller number of RPKs. Although the sample size of this study was small, the clinical data may be useful for guiding the decision-making regarding corneal transplantation in children, and we think this work is very important. We will involve more children in future studies to obtain more convincing results.

## Figures and Tables

**Table 1:** Indications for Primary and Repeat Penetrating Keratoplasty (PK) in Children.

	Number of Eyes (*n* = 30)	%
Indications for primary PK		
Congenital corneal opacity	26	86.67
Peters anomaly	16	53.33
Scleral cornea	4	13.33
Congenital aniridia	1	3.33
Unclear	5	16.67
Acquired corneal opacity	4	13.33
Bullous keratopathy	2	6.67
Corneal ulcer	1	3.33
Post-traumatic leukoma	1	3.33
Indications for repeat PK		
Vascularized corneal scar	15	50
Corneal ulcer	8	26.67
Bullous keratopathy	7	23.33

**Table 2:** Complications, Management, and Outcomes of RPK in Children.

Complications *	Number (N)	Management (N)	Outcomes (N)	
			Clear ^#^	Opacity
Rejection	Single * (4)	Intensive corticosteroid ^†^ (4)	1	13
	Compound * (10)	Intensive corticosteroid (6)/Unclear (4)	0	4
Epithelial defect	Single (2)	Bandage contact lens (1)/Medicine ^‡^ (1)	1	1
	Compound (9)	Bandage contact lens (2)/Medicine ^‡^ (3)/	1	8
		Tarsorrhaphy (2)/ AMT ^$^ (1)/Conjunctival flap (1)		
Glaucoma	Single (1)	Medicine (1)	1	0
	Compound (7)	Medicine(6)/Cyclocryotherapy(1)	0	7
Cataract	Single (3)	Surgery (2)/No surgery (1)	2	1
	Compound (4)	Surgery (2)/No surgery (2)	1	3
Corneal ulcer/erosion	Single (1)	Medicine (1)	0	1
Retro-corneal membrane	Compound(2)	Surgery(1)	0	1
		No surgery(1)	0	1
Hyphema	Compound(2)	Surgery(1)/No surgery(1)	0	2
Primary graft dysfunction	Compound(1)	-	0	1
Endothelial dysfunction ^&^	Compound(1)	-	0	1

* More than one complication may occur in the same eye. Single: only one complication occurred, Compound: two or more complications occurred. ^#^ Six cases with a postoperative complication had clear regrafts. Among them, one case had a compound complication of a cataract and epithelial defect, and the other five cases had a single complication. ^†^ Additional three doses of prednisolone acetate ophthalmic suspension 1%, which were administered 5 min apart in the morning, or an additional subconjunctival injection of 20 mg triamcinolone acetonide one to two times was prescribed. ^‡^ Artificial tears without preservative. ^$^ AMT Amino membrane transplantation. ^&^ Endothelial dysfunction secondary to intraocular surgery after RPK.

**Table 3:** Analysis of the Risk Factors That Could Affect the Survival of RPK in Children.

Characteristics	Hazard Ratio	95% Confidence Interval (CI)	p *
Age at surgery (m)			
>60	1		
≤60	5.210	1.505–18.036	0.009
Corneal vascularization			
No	1		
Yes	3.793	1.257–11.444	0.018
Postoperative epithelial defect			
No	1		
Yes	3.527	1.078–11.543	0.037

* *p* value was based on Cox regression.

## Data Availability

The data are available upon request from the corresponding author.
